# A Short Report on Melanocyte/Melanoma Culture, Senescence, and Reproducibility

**DOI:** 10.1111/pcmr.70084

**Published:** 2026-03-10

**Authors:** Lionel Larue, ESPCR Working Group, Duarte C. Barral, Veronique Delmas, Sara Egea‐Rodriguez, Daniel Aldea, Heather C. Etchevers, Marie‐Dominique Galibert, Robert N. Kelsh, Luisa Lanfrancone, Michele Madigan, Pedro Moura‐Alves, Richard M. White, Anja Bosserhoff

**Affiliations:** ^1^ INSERM U1021, Normal and Pathological Development of Melanocytes, Institut Curie PSL Research University Orsay France; ^2^ CNRS UMR3347 Paris‐Sud University, University Paris‐Saclay Orsay France; ^3^ iNOVA4Health, NOVA Medical School, Faculdade de Ciências Médicas, NMS, FCM Universidade NOVA de Lisboa Lisboa Portugal; ^4^ Department of Dermatology and Allergy, University Hospital LMU Munich Munich Germany; ^5^ INSERM, Marseille Medical Genetics, U1251, Institut MarMaRa Aix Marseille University Marseille France; ^6^ Department of Molecular Genetics and Genomics, CNRS, INSERM, IGDR (Institut de Génétique et Développement de Rennes)—UMR6290, ERL U1305—Hospital University of Rennes Univ. Rennes Rennes France; ^7^ Department of Life Sciences University of Bath Bath UK; ^8^ Department of Experimental Oncology IEO, European Institute of Oncology IRCCS Milan Italy; ^9^ Optometry and Vision Science UNSW Sydney Sydney Australia; ^10^ Save Sight Institute Sydney University Sydney Australia; ^11^ i3S, Instituto de Investigação e Inovação em Saúde Universidade do Porto Porto Portugal; ^12^ IBMC, Instituto de Biologia Molecular e Celular Universidade do Porto Porto Portugal; ^13^ Ludwig Cancer Research, Nuffield Department of Medicine University of Oxford Oxford UK; ^14^ Institute of Biochemistry FAU Erlangen‐Nürnberg Erlangen Germany

## Abstract

At the 2025 ESPCR (*European Society for Pigment Cell Research*) meeting in Erlangen, a workshop on “Pigment Cell Models: Sensitivity, Innovation, and the Challenges of Cell Culture” brought together researchers to discuss technical, methodological, and reproducibility issues in culturing melanocytes, keratinocytes, fibroblasts, and melanoma cells. The discussion between experts in the field highlighted key and recurrent pitfalls affecting experimental outcomes, including low‐density seeding, temperature fluctuations, over‐passaging, and mycoplasma contamination, as well as sources of variability arising from media composition, batch effects, and environmental conditions. Importantly, the workshop distinguished between practices supported by evidence and consensus‐based guidance derived from collective expert experience. Species‐ and donor‐specific differences, especially between human, mouse, and zebrafish melanocyte models, were identified as additional major determinants of experimental variability. Emerging systems, including human and mouse pluripotent stem cell (PSC)‐derived melanocytes, as well as avian and zebrafish melanoma lines, were discussed for their complementary mechanistic and translational value. Overall, the workshop concluded that transparent documentation, explicit reporting standards, and shared best practices are essential to improve reproducibility and further advance pigment cell research.

## Key Points

1. *Workshop focus (ESPCR 2025, Erlangen)*: International experts identified recurrent technical and methodological pitfalls in melanocyte, keratinocyte, fibroblast, and melanoma cell culture that undermine reproducibility.
2. *Major technical vulnerabilities*: Low‐density seeding, temperature fluctuations, over‐confluence/over‐passaging, and mycoplasma contamination significantly alter pigmentation, proliferation, metabolism, and differentiation state.
3. *Media and environmental variability*: Differences in media formulation, batch effects, proprietary supplements, oxygen tension, pH, CO_2_, and handling practices introduce substantial inter‐laboratory variability.
4. *Evidence vs. expert consensus*: The workshop clearly distinguished experimentally validated practices from consensus‐based guidance derived from collective experience in the pigment cell field.
5. *Species‐ and donor‐specific differences*: Human, mouse, and zebrafish melanocyte models differ in telomere biology, senescence mechanisms, microenvironmental context, and differentiation behavior, strongly influencing experimental interpretation.
6. *Emerging complementary models*: PSC‐derived melanocytes, zebrafish and avian melanoma lines, and large mammal systems expand mechanistic and translational opportunities but introduce additional standardization challenges.
7. *Planning principle*: Experimental design should begin at least two passages prior to assays (“two passages before” principle) to stabilize cultures and improve reproducibility.
8. *Documentation over rigid standardization*: Transparent reporting of cell origin, passage history, media composition, environmental conditions, and quality control measures is more impactful than imposing a single universal protocol.
9. *Community‐driven reproducibility*: Shared reporting standards, open protocol platforms, and explicit disclosure of variability are essential to strengthen robustness and comparability in pigment cell research.

## Graphical abstract

Culture phenotype is determined by the balance between variable physiology and implemented stability measures, ensuring robust and reproducible pigmentation, proliferation, metabolism, and differentiation data.

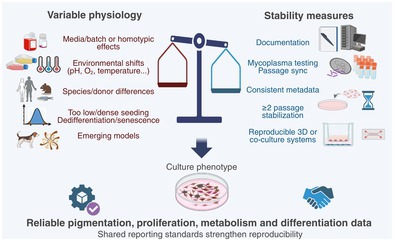

**Abbreviations**
α‐MSH: alpha melanocyte‐stimulating hormone
ATCH: adrenocorticotropic hormone
bFGF: basic fibroblast growth factor, also known as FGF2
BPE: bovine pituitary extract
CDK4: cyclin‐dependent kinase 4
CDKN2: cyclin‐dependent kinase inhibitor 2
DMEM: Dulbecco's Modified Eagle Medium
DMEM/F‐12: Dulbecco's Modified Eagle Medium: Nutrient Mixture F‐12
ESCs: embryonic stem cells
ESPCR: European Society for Pigment Cell Research
FBS: fetal bovine serum
hTERT: human telomerase reverse transcriptase
iPSCs: induced pluripotent stem cells
MCDB 153: molecular, cellular, and developmental biology medium 153
MITF: microphthalmia‐associated transcription factor
PSCs: pluripotent stem cells
PMA: phorbol 12‐myristate 13‐acetate, also called TPA or 12‐O‐tetradecanoylphorbol‐13‐acetate
RPMI 1640: Roswell Park Memorial Institute Medium 1640
SMAD: suppressor of mothers against decapentaplegic
SNP: single nucleotide polymorphism
WNT: wingless/integrated

## 1 Introduction: Workshop Context and Purpose

Research on melanocytes, their progenitors, and neighboring cells such as keratinocytes and fibroblasts faces specific persistent technical and methodological challenges. Although these cell types are widely used in experimental dermatology, developmental biology, and cancer biology, laboratories often encounter persistent issues related to standardization, reproducibility, and documentation of cell culture protocols. These challenges are exacerbated when studying melanoma, where cellular behavior can vary greatly as a function of the patient itself, cell line, passage history, and culture conditions. Differences in culture media composition, growth conditions, passage number, and handling practices can lead to significant variability in experimental outcomes, complicating reproducibility across laboratories.

This 2025 ESPCR workshop was designed to address these issues by providing a forum for discussion and knowledge‐sharing. The objectives were: (i) to identify and highlight technical pitfalls commonly encountered in the culture and use of melanocytes, keratinocytes, fibroblasts and melanoma primary cultures and cell lines, (ii) to explore alternative models and approaches, including 3D culture systems, organoids, and co‐culture techniques that may provide more physiologically relevant insights, and (iii) to promote improved reproducibility in melanocyte and melanoma research respecting phenotypic variability, by encouraging rigorous documentation, transparent method reporting, and development of common standards.

By bringing together researchers with diverse expertise, the workshop generated a set of recommendations and guidance to improve reliability, reproducibility, and interpretability in pigment cell research.

## 2 Key Challenges in Cell Culture

The culture of skin cells presents unique challenges that directly impact experimental outcomes. Unlike immortalized or transformed cell lines, primary cells retain many features of their in situ and in vivo environment, making them sensitive to rapid deviations in handling, culture conditions, and medium composition. Below, we outlined the most common sources of variability and experimental failure. Importantly, we differentiate between practices supported by evidence and consensus‐based, collective expertise, as only a minor number of cell culture handling techniques have been optimized in controlled experimental studies.

### 2.1 Technical Pitfalls

As usual in molecular and cellular biology, it is important to strictly follow a protocol. Protocols for culturing murine, as well as human melanocytes and melanomas derived from patient tissue or tumor grown in immunocompromised mice have been published (Aktary et al. 2023; Berlin et al. 2012; Eberle et al. 2010; Gallagher et al. 2011; Goff et al. 2023; Licciulli et al. 2011). These protocols do not pose any major technical difficulties apart from monitoring the cultures every day or two. However, technical pitfalls are often encountered, especially for beginners.

#### 2.1.1 Low‐Density Seeding

A common practice used to avoid weekend maintenance is to seed cells at low density on Fridays (e.g., below ~2–5 × 10^3^ cells/cm^2^). Although this may be convenient, sparse cultures place cells under significant stress, triggering selective pressures that can accelerate genetic and phenotypic drift, thus affecting pigmentation and proliferation status (Godwin et al. 2014), and expert consensus from workshop participants. The result is a population that diverges from the original culture, reducing both comparability and reproducibility. Similarly, thawing of skin‐derived cells must be performed in a suitably sized culture dish to achieve an initial density (confluence) of at least 50% of the total area. Although this issue is clearly relevant for human or mouse cells, zebrafish melanoma cells cultured under low‐density conditions appear particularly sensitive and poorly tolerate sparse seeding (expert consensus opinion from workshop participant R). White, based on experience from Heilmann et al. (2015). Although the exact reasons for such density differences are not well understood, it is important to understand this tolerance for each cell line both within and across species.

#### 2.1.2 Temperature Inconsistencies

The use of cold media or abrupt temperature shifts can impair cellular growth kinetics. Beyond growth delay, these fluctuations alter cellular metabolism, affect protein folding, and destabilize melanogenic pathways, further adding variability in responses and complicating the interpretation of results (Kim et al. 2003). However, some media components, particularly bFGF (Chen et al. 2012), are increasingly labile as temperature increases, with shorter half‐lives. The solution is to make a small working aliquot of reconstituted medium stock that could last for a few days, pre‐warm it for the minimum time to reach temperature and regularly replace it with fresh stock. Alternatively, the serum‐supplemented medium can be kept at 4°C, and the growth factors (thawed from frozen aliquots) added just before cell splitting.

#### 2.1.3 Over‐Confluence and Over‐Passaging

Cultures that reach excessive confluence or are propagated for too many passages often lose their defining characteristics, leading to failed or misleading experiments. Careful monitoring of passage numbers and confluence thresholds is therefore essential (Poumay and Pittelkow 1995). Do not forget to document the morphology of your cells by taking pictures during the passages and to freeze amplified cells at a predetermined passage number to stock enough cells for the entire experiment. This allows clear observation of any morphological changes (if they occur) and return to earlier passages. This is important not only for documenting any morphological changes but also because in recent human skin studies, melanocyte morphology is correlated with cell function. Specifically, researchers from independent groups have identified distinct populations of MITF‐M‐expressing melanocytic cells based on their shape and marker co‐expression. Functionally mature melanocytes are often pigmented and poly‐dendritic (“star‐shaped”), although morphology varies with species, media composition, and differentiation state. This could be based on phenotypic variability induced by the impact of density, where cells with an immature, bipolar morphology that show almost no pigmentation express some neural crest stem cell (NCSC) markers and possess multilineage differentiation potential. This strongly suggests that the bipolar shape corresponds to an NCSC‐like cell serving as an interfollicular reservoir for melanocytes, whereas the dendritic shape marks a fully melanogenic, differentiated state (Brombin and Patton 2024; Klar et al. 2017; Michalak‐Mićka et al. 2022; Tobin and Bystryn 1996).

In conclusion, collectively, these findings demonstrate that extended culture duration and excessive confluence can profoundly alter melanocyte morphology and pigmentation, with direct consequences for functional readouts and experimental validity. Accordingly, there is broad consensus that researchers should systematically document cell morphology at every passage, freeze cell stocks at predetermined passage numbers (and use this stock for the entire set of experiments), and actively monitor differentiation status; distinguishing mature, poly‐dendritic melanocytes from immature, bipolar cells to ensure experimental reproducibility and biological relevance.

#### 2.1.4 “Two Passages Before” Principle

After discussion, we concluded that to ensure reproducibility, experimental planning should begin at least two passages prior to the actual experiment. This stabilization period allows cells to recover from stress and ensures that the culture conditions are consistent with the planned assay. However, in the case of primary cultures derived directly from the patient, where significant phenotypic changes can occur during extended culture, it is suggested to limit their time in culture to a single passage if possible (expert consensus from workshop participants).

### 2.2 Quality Control and Standardization of Cell Cultures

#### 2.2.1 Mycoplasma and Contamination

Cultures generated from skin are especially prone to mycoplasma contamination. These can strongly influence, for example, cell metabolism and growth kinetics (Carrington and Ward 1988; Gedye et al. 2016). Testing for mycoplasma contamination should be performed regularly and is easily done using commercial PCR kits on aliquots of conditioned media. If positive, cells should be discarded and the assays redone from a new batch. If this is not feasible, the cells must be appropriately treated, experiments delayed, and only re‐initiated once the test is negative. In all cases, any mycoplasma treatment must be clearly reported in the Materials and Methods section of publications.

#### 2.2.2 Cell Characterization

A strict and continuous control of cell identity is necessary. Establish methods to regularly confirm the identity of the cells used and possible cross‐contamination. Also consider including regular short tandem repeat (STR) genotyping to verify cell lines (Almeida et al. 2019; Capes‐Davis et al. 2019). This control is strongly recommended for experimental outcomes and also requested by publishers and/or by funding institutions to ensure reproducibility.

#### 2.2.3 Nomenclature

When a primary culture of melanocytes generates a new cell line, it should be named as clearly as possible. For example [*Mc‐Tyr::Nras* ^Q61R/°^; *TyrCre*/°; *Pten* ^f/+^; mouse number] or any other unique and unambiguous naming system. Consign the naming rationale to a file alongside your culture records and increment the version if it evolves over time (expert consensus from workshop participants).

### 2.3 Media and Supplements

#### 2.3.1 Brand and Batch Variability

Even when nominally identical (e.g., RPMI 1640), media formulations can diverge significantly between different suppliers and batches in terms of ion and nutrient composition and buffering capacity. Such differences can have profound consequences on various aspects of cellular function including alterations in transcription factor activity, downstream signaling, or pigmentation.

Many groups cultivate melanocytes in a specialized commercial culture medium (a defined base medium + a melanocyte growth supplement) or in many different generic cell culture media (DMEM, RPMI, DMEM:F12, MCDB153, McCoy's 5A) modified with specific supplements including fetal bovine serum, PMA, cholera toxin, bFGF, endothelins, stem cell factor, and insulin, Wnt3a or pathway agonists such as CHIR99021 (Bizik et al. 1996; Gilchrest et al. 1984; Hirobe et al. 2016; Jee et al. 1994; Kim et al. 2005; Kobori et al. 2023; Kormos et al. 2011; Mansur et al. 1988; Medrano et al. 1994; Padma and Bhat 2016; Szabad et al. 2007; Zhu et al. 2004; Cohen et al. 2023; Goff et al. 2023; Licciulli et al. 2011). This diverse range of media and supplemental agents has been employed to study melanocyte physiology. Of course, harmonization in this domain would be desirable to better compare results across studies.

#### 2.3.2 Handling Practices

Proper handling of media and supplements is critical for maintaining cell viability, responses, and phenotypes (expert consensus from workshop participants): (i) *Light protection*: Media exposed to light can undergo photodegradation, reducing nutrient quality and generating reactive by‐products (e.g., tryptophan metabolites; Oberg et al. 2005). (ii) *Pre‐warming*: Media aliquots for changes should be equilibrated to 37°C before use; incomplete warming or prolonged exposure to room temperature destabilizes buffering capacity and induces cellular stress or irreversible shock. (iii) *CO*_*2*_ *control*: Maintaining the correct CO_2_ concentration during handling ensures proper pH buffering. (iv) *Aliquoting labile factors*: Many supplements degrade rapidly if repeatedly thawed. Aliquoting into single‐use vials stored at the appropriate temperature extends activity and consistency. (v) *Minimizing passage‐induced stress*: Sensitive cell types often respond poorly to abrupt changes in their microenvironment during passaging. Supplementing the fresh medium with a portion of conditioned medium from the previous culture can provide beneficial soluble factors such as growth‐promoting cytokines, extracellular matrix components, and metabolic by‐products, that help buffer the transition and support post‐passage recovery.

#### 2.3.3 Defined vs. Proprietary Formulations

Earlier melanocyte culture protocols relied on named supplements such as cholera toxin, growth factors, ACTH, or α‐MSH to modulate proliferation and pigmentation. By contrast, many modern commercial formulations are proprietary and undisclosed, raising concerns about unintended pathway activation or suppression. Such hidden variables introduce biases that may not be apparent until they affect experimental results.

#### 2.3.4 Environmental Factors

The main environmental factors that can be easily controlled are pH, temperature, and CO_2_. Oxygen levels can also be regulated; however, doing so typically requires specialized and costly equipment. (i) *pH regulation*: The skin surface exhibits site‐dependent pH variation, often more acidic (pH ~4.1–5.8) than standard culture conditions (pH ~7.1–7.5; Brooks et al. 2025). This mismatch can alter enzymatic activity, barrier function, and stress responses. (ii) *Temperature differences*: Human skin typically functions at 32°C–36°C, whereas standard incubators maintain a temperature of 37°C. This seemingly minor difference can influence enzyme kinetics, cell cycle progression, and responses to stress. (iii) *CO*_*2*_ *fluctuations*: Variability in CO_2_ levels directly impacts buffering capacity and pH, thereby influencing pigmentation, cell signaling, and overall culture stability. Careful calibration and monitoring of incubators is critical to reduce these fluctuations. (iv) *Oxygen levels*: In vivo epidermal oxygen is relatively low (1%–5%), reflecting the diffusion barrier of the stratum corneum. Cultures grown in atmospheric oxygen (~20%) experience hyperoxic stress (Carreau et al. 2011; Weidmann et al. 2017, eye melanocytes), which may accelerate oxidative damage, slow doubling times, and alter pigmentation pathways. Mouse (B16‐F10) and human (SK‐MEL‐28) melanoma cell lines change behavior and gene expression as a function of oxygen levels (Cheli et al. 2012; Loftus et al. 2017).

Factors influencing in vivo conditions in humans, mice, and other species across different skin areas, other pigmented tissues, sexes, and/or ages remain poorly understood. However, any deviations from these conditions are likely to increase stress on cultured cells, making them more sensitive to other stressors and affecting the reproducibility of the culture (expert consensus from workshop participants). Over the long term, targeted measurement of in vivo conditions and direct testing of melanocyte and keratinocyte cultures under more physiological conditions would be invaluable for identifying the conditions most crucial to driving reproducibility.

## 3 Melanocyte Models and Senescence

### 3.1 Human vs. Mouse vs. Zebrafish

A major difference between humans and mice is that mouse skin melanocytes are primarily located in hair follicles rather than throughout the epidermis. Zebrafish melanocytes are often contained within scale structures or, alternatively, in the dermis. This implies that melanocytes from different species are exposed to different microenvironments (Hou et al. 2006; Mort et al. 2015).

#### 3.1.1 Telomere Length

Mouse melanocytes have longer telomeres than human melanocytes, which allows them to resist senescence for extended periods. This often requires additional manipulation, such as the introduction of human telomerase reverse transcriptase (hTERT), to achieve immortalization. In contrast, mouse melanocytes have shorter telomeres and bypass senescence more readily under certain genetic modifications (Goff et al. 2023). When melanocytes bypass senescence, it is recommended to trypsinize cells for passage gently. This has the advantage of eliminating senescent cells, which are more adherent and less resistant.

#### 3.1.2 Senescence Bypass Mechanisms

Mouse melanocytes can bypass senescence with spontaneous or targeted deletion of *Cdkn2a*, encoding p16 (Goff et al. 2025). Human melanocytes, however, generally require both inactivation of P16 (or expression of CDK4) and active telomere maintenance to achieve a similar effect (Gray‐Schopfer et al. 2007, 2006; Sviderskaya et al. 2003). This highlights species‐specific differences in senescence regulation.

#### 3.1.3 Genetic Drift in Cell Stocks

All cell cultures are susceptible to genetic drift over successive passages, a problem particularly addressed in the stem cell field (Andrews et al. 2022). Careful stock generation, freezing, accurate passage tracking, and routine authentication are crucial for maintaining experimental consistency (Ben‐David et al. 2018).

### 3.2 Hermes Cell Lines

Hermes cell lines are well‐characterized immortalized human melanocytes (e.g., Gray‐Schopfer et al. 2006). They are genetically manipulated to inactivate *CDKN2A* (affecting P16 and P14) and express hTERT. The main use for these cell lines is in long‐term experiments including studies of proliferation, signaling, and drug response. However, they present some limitations despite their utility; they may not fully recapitulate the phenotype of primary melanocytes, particularly regarding their differentiation state, epigenetic landscape, or senescence responses.

### 3.3 iPS‐ and ES Cell‐Derived Melanocytes

#### 3.3.1 Differentiation Protocols

Melanocytes can be generated from pluripotent stem cells (iPSCs and ESCs) through a stepwise differentiation process that recapitulates embryonic development (Kunisada et al. 2003; Liu et al. 2020; Pla et al. 2004): (i) *Inner cell mass to ectoderm*: Pluripotent cells are first guided toward the ectodermal lineage using defined culture conditions and signaling cues. (ii) *Ectoderm to neural crest cells*: The ectoderm differentiates into neuroepithelium and neural crest cells, which are characterized by the expression of a gene regulatory network including transcription factors such as Sox10, Foxd3, and Pax3. These multipotent progenitors give rise to several lineages (Etchevers et al. 2019) including melanocytes. (iii) *Fate restriction*: Neural crest cells are further specified into Schwann cell precursors and Mitf‐expressing melanoblasts, which can each mature into functional melanocytes (Colombo et al. 2022). Although it is relatively easy to maintain cells in neural crest or melanocytic states, melanoblasts are highly unstable and difficult to propagate before they spontaneously differentiate (expert consensus from workshop participants). Finally, mature melanocytes must express lineage‐specific markers, such as dopachrome tautomerase (Dct) and tyrosinase (Tyr), along with other pigmentation‐related enzymes that require copper as a cofactor; hence, copper supplementation is a key aspect of culture media. This step ensures the cells acquire functional characteristics like those of in vivo melanocytes (Peng et al. 2019). Finally, we should mention that choices like endothelin‐3 versus endothelin‐1 as an endothelin receptor type B (EdnrB) agonist are often historically driven rather than strictly mechanistic, highlighting the need for careful protocol rationale. To date, almost all ESC/iPSC protocols have used endothelin‐3 to induce melanocytes.

#### 3.3.2 Modulation Strategies of Human and Mouse iPS and ES Cells

The efficiency and fidelity of melanocyte differentiation can be enhanced by manipulating key signaling pathways and the cellular environment: (i) Wnt pathway activation promotes melanocyte specification and supports melanoblast proliferation (Cohen et al. 2023), (ii) inhibition of BMP‐mediated SMAD signaling facilitates neural crest induction and prevents alternative lineage differentiation (Mica et al. 2013), (iii) EdnrB agonists will favor outgrowth of mature melanocytes (Hyter et al. 2013; Lahav 2005), (iv) modulation of cAMP impacts the differentiation state of the melanocytes (Bang and Zippin 2021; Wang et al. 2022), and (v) trace element supplementation, such as copper, is essential for enzymatic activity of tyrosinase and proper pigmentation in differentiated melanocytes (Kim et al. 2023; Solano 2018).

#### 3.3.3 Species Differences

Human and mouse pluripotent stem cells show substantial differences in differentiation efficiency, lineage commitment, and final melanocyte phenotype. Human iPSCs often require longer differentiation times and additional signaling modulation compared with mouse cells. These species‐specific differences influence experimental design, reproducibility, and the interpretation of functional studies. Avian species vary in their efficiency of melanocytic differentiation as a function of both cell‐intrinsic and cell‐extrinsic factors (Jacobs‐Cohen et al. 2002). Even within species, there is considerable individual variation across ES or iPS cell lines in terms of efficiency of differentiation to melanocytes. Some can reach 100% melanocytes after 15–20 passages (Hosaka et al. 2019). This could be due to multiple factors including the experimenter, germline genetics (e.g., single nucleotide polymorphism—SNPs), or unwitting clonal selection during iPSC generation or subsequent passages.

#### 3.3.4 Applications and Limitations

iPSC‐ and ESC‐derived melanocytes provide a versatile platform for modeling human pigmentation, disease mechanisms (e.g., vitiligo, melanoma), drug screening, and gene editing studies. However, these cells may not fully replicate all aspects of adult melanocyte physiology such as the epigenetic landscape and long‐term senescence behavior. Furthermore, differentiation protocols are often time‐consuming and can vary significantly across different cell lines. Finally, iPS or ES cells from different human donors can vary widely in the speed at which they differentiate into mature melanocytes, often differing by weeks. This is likely due to germline differences across different patients (expert consensus from workshop participants).

### 3.4 Challenges (Expert Consensus From Workshop Participants)

#### 3.4.1 Senescence Induction Difficulties

In immortalized melanocyte lines, attempts to induce senescence with small molecules or stressors frequently fail or lead to cell death.

#### 3.4.2 Cell Source Limitations

Foreskin‐derived fibroblasts, keratinocytes, and melanocytes are commonly used because they are readily available and have high proliferative capacity. However, they may not accurately reflect adult tissue physiology or disease‐relevant states, and their use limits studies to male neonatal glabrous skin cells. Adult cutaneous melanocytes can be obtained from tissues removed during plastic surgeries, such as the abdomen or breast, providing a physiologically relevant source from both sexes. Nonetheless, in all cases, melanocytes are derived from specific body sites, which may influence their behavior and limit generalizability.

#### 3.4.3 Donor Variability

Age, sex, phototype, and tissue source of the donor cells can profoundly affect senescence onset, proliferation rates, and experimental outcomes. If using postmortem tissue (e.g., human eye tissue for uveal melanocytes or retinal pigment epithelium—RPE), details on the time elapsed since death and the cause of death will affect cell characteristics and experimental outcomes. Iris color may be used as an indicator of tissue pigmentation (e.g., eye melanocytes differ in pigmentation from donors with brown vs. blue irides). At this point, it is of interest to mention that protocols specifically for the culture of eye melanocytes, RPE and melanoma cells have been established (Chen et al. 2025; Song et al. 2025). Careful selection and characterization of donor cells are therefore crucial.

## 4 Reproducibility and Standardization

Reproducibility is a cornerstone of robust scientific research, yet it remains a significant challenge in cell‐based studies, particularly with normal melanocytes and melanoma models. Standardization is often considered the solution, but in practice, variability is inevitable and can be informative if carefully documented and interpreted. Variability should not be viewed solely as experimental noise but as biological information when appropriately documented.

### 4.1 Variability Across Labs

Even when using ostensibly identical cell lines, notable differences emerge between laboratories. For example, (i) *Gene expression and phenotype*: Melanocyte cultures exhibit inter‐ and intra‐laboratory variability in gene expression profiles, pigmentation levels, and cellular morphology. These differences can complicate comparisons and meta‐analyses, (ii) *Environmental sensitivity*: Melanocytes are acutely responsive to culture conditions including media composition, passage number, cell density, and even supplier‐specific reagents. Small deviations can lead to large functional differences, and (iii) *Melanoma cell line stability*: In contrast, melanoma‐derived lines tend to be more robust, showing consistent clustering in transcriptomic analyses across laboratories. This stability facilitates comparative studies but does not fully eliminate variability.

### 4.2 Reporting

Reporting standards for pigment cell culture studies need to be set up to give all relevant information in published studies. Based on the discussion in the workshop, we suggest the criteria featured in Table 1.

**TABLE 1:** Recommended reporting standards for pigment cell culture studies.

Cell identity and origin Cell type (e.g., primary melanocytes, melanoma cells, keratinocytes, fibroblasts) Species (human, mouse, zebrafish, avian, etc.) Tissue source and anatomical site (e.g., foreskin, trunk skin, hair follicle, eye/uvea) Developmental stage or age at isolation Sex of donor Phototype, pigmentation status, or relevant visible traits (e.g., iris color for ocular cells) Primary culture vs. established cell line Cell line name and origin Donor and sample metadata Relevant clinical information including disease status For postmortem tissue: postmortem interval and cause of death Ethical approvals and consent statements Culture history and passage information Passage number at the start and end of experiments Maximum passage number used Passage frequency and split ratios Duration of culture prior to experimentation Description of any stabilization period prior to assays (e.g., “two passages before” principle) Culture media and supplements Base medium name, manufacturer, and catalogue number Complete formulation including all supplements and growth factors Supplier, catalogue number, and lot/batch number (when known) Concentrations of key additives (e.g., bFGF, endothelins, PMA, cAMP modulators, and so forth) Environmental and handling conditions Incubator temperature CO_2_ concentration Oxygen level (normoxia vs. hypoxia) pH Light exposure considerations Media pre‐warming and handling practices Use of conditioned medium or feeder layers (if applicable) Cell density and confluence Seeding density (cells/cm^2^) Confluence at seeding, passaging, and experimentation Rationale for low‐ or high‐density culture Cell characterization and quality control Methods used to verify cell identity (e.g., STR profiling, genotyping) Frequency of authentication Mycoplasma testing method and frequency Description of any contamination treatment Morphological assessment criteria (with representative images where possible) Genetic and epigenetic status Key genetic modifications or mutations (e.g., BRAF, NRAS, CDKN2A status) Method of genetic manipulation (e.g., CRISPR, viral transduction) Telomerase or senescence‐bypass strategies Known limitations of genetic stability Differentiation state and functional validation Markers used to define melanocytic identity or differentiation stage Pigmentation assays or functional readouts Evidence supporting maturity or lineage commitment Distinction between evidence‐based validation and expert consensus

## 5 Alternative Models and Species

Understanding melanocyte biology and melanoma progression benefits from exploring models beyond human cell lines. Different species and model systems offer unique insights, each with strengths and limitations.

### 5.1 Fish and Amphibians

(i) Zebrafish ( *Danio rerio* ): Widely used for studying melanocyte development, pigmentation patterns, and keratinocyte interactions due to transparent embryos and genetic tractability. Multiple well‐characterized zebrafish melanoma cell lines (e.g., ZMEL1, ZCREST1) have been generated and have remained stable for over 10 years (Heilmann et al. 2015; Kaufman et al. 2016). These have been extensively characterized at genomic, transcriptomic, and phenotypic levels. They readily give rise to aggressive and metastatic melanoma when transplanted back into fish (Campbell et al. 2021; Kim et al. 2017) but unfortunately do not produce liver metastasis (van den Bosch et al. 2024). Single‐cell RNA‐seq of the ZMEL1 line (both in vitro and in vivo) has shown that it exhibits the same 4–5 key cell states described in human melanomas (i.e., neural crest, melanocytic, mesenchymal, etc.), suggesting that it closely recapitulates key aspects of the disease (Hunter et al. 2021). To date, no normal melanocyte counterparts have been successfully established in cell culture, although this could, in principle, be achieved by immortalizing them with SV40 or TERT. (ii) *Medaka, cavefish, killifish, salmon*: These and other species allow investigation into alternative pigment cell types (Bjørgen and Koppang 2024; Thorsen et al. 2006), evolutionary adaptations (e.g., troglomorphic depigmentation; Zhang et al. 2025), and aging‐related pigment changes. (iii) *Xenopus laevis* and *Xenopus tropicalis* have been considered less suitable for melanocyte or melanoma studies with translational relevance to humans, largely due to limited applicability to genetic and cancer‐focused research. However, the technical advantages can outweigh these considerations, as in a recent *BRAF* ^*V600E*^‐targeted *mitf* locus knock‐in model (Ran et al. 2025). A general caveat to using fish and amphibian lines is that they grow at considerably different temperatures, often in the range of 20°C–28°C. This has not been systematically studied but should be considered when comparing to humans.

### 5.2 Large Mammal Models

(i) *Pig, dog, horse*: These offer closer physiological and anatomical relevance to human skin than rodents. Certain dog breeds develop mucosal melanoma with genetic features resembling human disease, making them a valuable source of cell lines and translational insights (Gillard et al. 2014; Okauchi et al. 2025; Segaoula et al. 2018). Pigs and horses can be helpful in pigmentation and skin biology studies (Travis et al. 2015), though logistical and cost considerations limit widespread adoption. Both have races predisposed to certain types of melanoma, from which cell lines can be derived (Brodesser et al. 2025; Chapman et al. 2009). (ii) *Primates*: Non‐human hominid research is largely restricted by ethical considerations. In historic research colonies, chimpanzees at least showed little evidence of spontaneous melanoma, limiting utility (Brown et al. 2009).

### 5.3 Protocol Decisions and Ethical Considerations

There is a reasonably high degree of standardization across protocols; however, key decisions such as the choice of differentiation cues and the selection of species continue to reflect historical precedent as well as ethical, regulatory, and practical constraints. These factors shape which species are feasible for research, influence experimental design, and affect translational relevance, as highlighted by expert consensus from workshop participants.

## 6 Standardization vs. Documentation

Exhaustive documentation is far more valuable than a single “gold standard,” as it is often fruitful to revisit older work when standards change. Critical factors include passage number, genetic status, gas and medium composition, donor source, and culture temperature. All these need to be clearly stated in publications (see Table 1) to set high standards in our area of research. Embracing variability can reveal biological insights, and community platforms like https://protocols.io and https://bio‐protocol.org/exchange/ facilitate the dissemination of shared, forked, and evolving protocols (expert consensus from workshop participants).

## 7 Conclusion

Research on melanocytes and melanoma is shaped by biological fragility, species‐specific differences, and methodological constraints. Key points include: (i) *Melanocyte culture fragility*: Highly sensitive to media composition, handling practices, donor source, and microenvironmental factors. (ii) *Passage history*: Proper planning ensures cells are in an optimal state, ideally monitored at least two passages ahead of experimentation. (iii) *Species differences*: Human and mouse melanocytes differ in pH tolerance, oxygen sensitivity, telomere dynamics, senescence, and differentiation capacity. (iv) *Documentation over standardization*: Transparent reporting of all culture conditions, passage history, and media composition is essential for reproducibility. (v) *Alternative models to complement human studies*: Many vertebrates and ES or iPSC‐derived systems provide complementary mechanistic insights and translational relevance. (vi) *Defined media to improve reproducibility*: Reliance on proprietary commercial formulations (“black boxes”) can introduce long‐term variability and reduce comparability across labs. (vii) *Community engagement toward strengthening science*: Sharing protocols including challenges and failures, fosters collective learning, and improvements in reproducibility.

## Author Contributions

**Lionel Larue:** conceptualization, methodology, writing – original draft. **Duarte C. Barral:** methodology, writing – review and editing. **Veronique Delmas:** methodology, writing – review and editing. **Sara Egea‐Rodriguez:** methodology, writing – review and editing. **Daniel Aldea:** methodology, writing – review and editing. **Heather C. Etchevers:** methodology, writing – review and editing. **Marie‐Dominique Galibert:** methodology, writing – review and editing. **Robert N. Kelsh:** methodology, writing – review and editing. **Luisa Lanfrancone:** methodology, writing – review and editing. **Michele Madigan:** methodology, writing – review and editing. **Pedro Moura‐Alves:** methodology, writing – review and editing. **Richard M. White:** methodology, writing – review and editing. **Anja Bosserhoff:** conceptualization, methodology, writing – original draft.

## Conflicts of Interest

The authors declare no conflicts of interest.

## Data Availability

The data that support the findings of this study are available on request from the corresponding author. The data are not publicly available due to privacy or ethical restrictions.
